# Concurrent Wagner syndrome and retinopathy of prematurity

**DOI:** 10.1016/j.ajoc.2025.102392

**Published:** 2025-07-22

**Authors:** Landon J. Rohowetz, David W. Redick, Kenneth C. Fan, Audina M. Berrocal

**Affiliations:** Department of Ophthalmology, Bascom Palmer Eye Institute, Miller School of Medicine, University of Miami, 900 NW 17th Avenue, Miami, FL, 33136, USA

**Keywords:** Inherited retinal disease, Retinopathy of prematurity, Wagner syndrome

## Abstract

**Purpose:**

To describe the clinical features of a patient with Wagner syndrome and a history of retinopathy of prematurity (ROP).

**Observations:**

A 23-year-old Hispanic male was referred for retina evaluation. The patient had a history of regressed ROP in both eyes that was never treated. The patient was born at 26-weeks gestational age and received supplemental oxygen as a neonate. He also reported a history of strabismus treated with surgery at 6 years of age. Best-corrected visual acuity was 20/30 in both eyes. Manifest refraction revealed −8.25 diopters of myopia in both eyes. Posterior segment examination demonstrated vitreous syneresis, a regressed temporal ridge, pigmented lattice degeneration, and atrophic holes in both eyes. Fluorescein angiography revealed temporal small vessel leakage and staining in both eyes without exudation. Electroretinography demonstrated reduced a- and b-wave amplitudes in both eyes. Optical coherence tomography of the macula revealed a blunted foveal contour in both eyes. Genetic testing revealed a heterozygous versican (VCAN) mutation: c.425C > T (p.Thr142Met).

**Conclusion and importance:**

The current case represents a rare combination of diseases and underscores the importance of considering concurrent retinal and vitreoretinal conditions in patients with a history of ROP.

## 1 Introduction

Wagner Syndrome is a rare genetic vitreoretinopathy characterized by myopia, cataract, strabismus, and chorioretinal atrophy.^1^ The disease is associated with mutations in the versican (VCAN) gene, which encodes for an extracellular matrix proteoglycan involved in cell proliferation, adhesion, migration, and angiogenesis.^1^, ^2^, ^3^, ^4^, ^5^ It is hypothesized that wild type versican prevents collagen fibrils from adhering to each other, giving the vitreous its gel-like properties. In Wagner syndrome, dysfunction of versican leads to early liquefaction of the vitreous and development of a thickened posterior hyaloid which in turn may lead to an increased risk of retinal detachment.^1^^,^^2^^,^^5^

Retinopathy of prematurity (ROP) is characterized by abnormal retinal vascular development in the neonatal period.^6^ In the absence of treatment, ROP can progress to neovascularization with resultant vitreoretinal traction and retinal detachment.^6^^,^^7^ Complications of ROP are not typically driven by vitreous abnormalities so the effects of coexisting ROP and Wagner syndrome are unclear. There is currently very limited data on ROP in the presence of other vitreoretinal diseases. In this case report, we present a patient with Wagner syndrome and a history of ROP.

## 2 Case report

A 23-year-old Hispanic male was referred for retina evaluation. He was born at 26-weeks gestational age and received supplemental oxygen as a neonate. The patient had a history of regressed ROP in both eyes that was never treated. He underwent heart surgery as an infant and strabismus surgery at the age of 6 years.

**Fig. 1:**
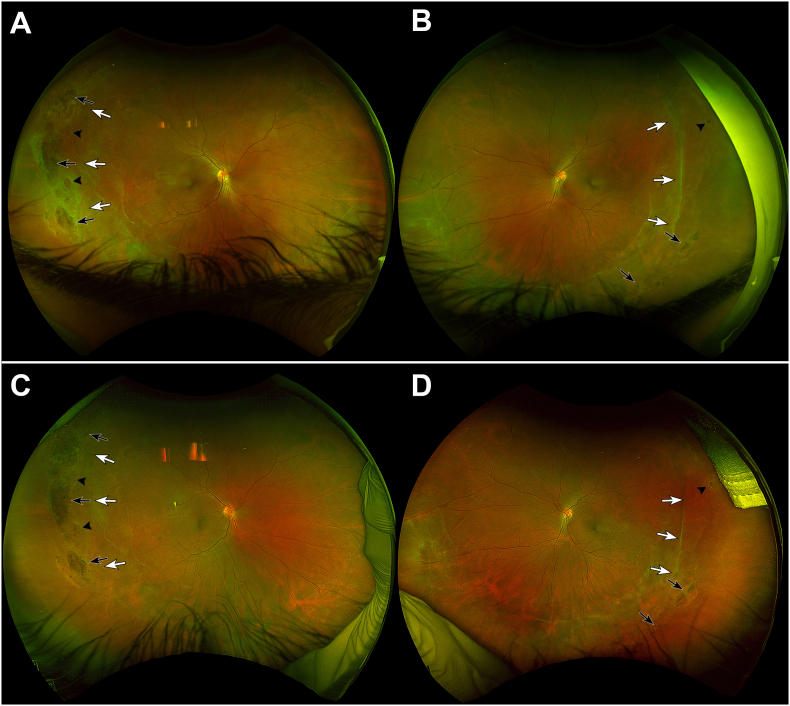
Fundus photography A 23-year-old male born at 26-weeks gestational age with a history of regressed retinopathy of prematurity presented to the clinic for retina evaluation. Fundus photography of the right (A) and left (B) eyes at presentation demonstrated vitreous syneresis, a regressed temporal ridge (white arrows), pigmented lattice degeneration (black arrows), and atrophic holes (arrowheads) in both eyes. Genetic testing revealed the presence of a versican (VCAN) mutation consistent with concurrent Wagner syndrome. Fundus photography 10 years later demonstrated stable vitreous syneresis, regressed temporal ridges (white arrows), and atrophic holes (arrowheads) in the right (C) and left (D) eyes.

**Fig. 2:**
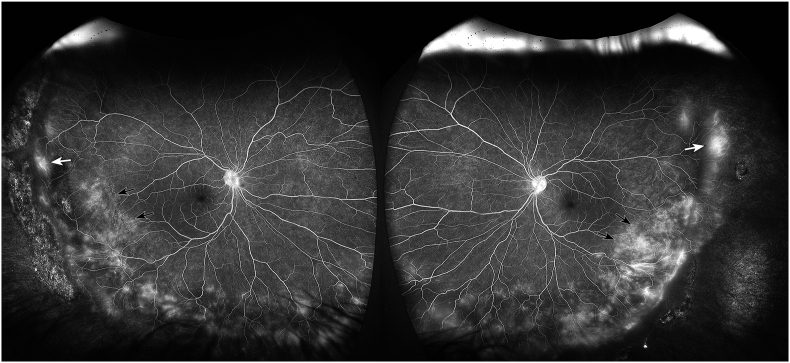
Fluorescein angiography Late-phase fluorescein angiography of a 23-year-old male born at 26-weeks gestational age with a history of Wagner syndrome and regressed retinopathy of prematurity revealing zone III small vessel leakage (white arrows) and temporal staining in zones II and III (black arrows) in the right (A) and left (B) eyes.

On initial presentation, the patient's best-corrected visual acuity was 20/30 in both eyes. Manifest refraction was −8.25 + 1.00 at 88° in the right eye and −8.25 + 1.00 at 40° in the left eye. Intraocular pressure was 17 mmHg in the right eye and 18 mmHg in the left eye. Strabismus examination demonstrated an esotropia of 20 prism diopters at near fixation and 14 prism diopters at distance. Extraocular movements were full in both eyes. Anterior segment examination revealed a small faint anterior stromal scar in the cornea of the right eye and was unremarkable in the left eye. Posterior segment examination demonstrated vitreous syneresis, a regressed temporal ridge, pigmented lattice degeneration, and atrophic holes in both eyes (Fig. 1A and B). Fluorescein angiography revealed small vessel temporal leakage in zone III and temporal staining in zones II and III in both eyes without exudation (Fig. 2A and B). Optical coherence tomography (OCT) of the macula showed fovea plana in both eyes. Fundus autofluorescence demonstrated hypoautofluorescence at the areas of lattice degeneration and along the regressed ROP ridge temporally in both eyes. Conventional full-field electroretinography revealed diminished scotopic and photopic a- and b-wave amplitudes in both eyes.

Invitae genetic testing (San Francisco, CA) revealed a heterozygous VCAN mutation: c.425C > T (p.Thr142Met). This variant was identified as ‘probably damaging’ using PolyPhen-2 polymorphism phenotyping, consistent with a diagnosis of Wagner syndrome. Heterozygous mutations in crumbs homolog 1 (CRB1) c.1465G > A (p.Glu489Lys) and elongation of very long chain fatty acids protein 4 (EVOVL4) c.931G > A (p.Ala311Thr) were also found. The patient has been followed for 10 years with stable visual acuity and examination findings (Fig. 1C and D).

## 3 Discussion

Wagner Syndrome is a rare inherited vitreoretinopathy associated with mutations in the VCAN gene.^1^ Clinical features of Wagner syndrome include myopia, cataract, retinal pigment epithelial changes, degenerative vitreous abnormalities, and an increased risk of retinal detachment.^1^^,^^2^^,^^5^ ROP is a retinal vascular disorder in premature infants that affects the development of the retina and retinal blood vessels.^6^^,^^7^ Sequelae of ROP can develop from cicatricial vitreoretinal changes after regression of the disease.^7^^,^^8^ Common findings include high myopia, retinal telangiectasias, pigmentary changes, lattice-like degeneration, macular dragging, fovea plana, retinal tears, and rhegmatogenous and tractional retinal detachments.^8^^,^^9^

The role of coexisting vitreoretinal disease in the presence of ROP in not well understood. It is possible that an underlying retinal or vitreoretinal disease may confound the natural progression of ROP. While many adults with a history of ROP demonstrate stability of their condition with older age, the presence of concurrent vitreoretinal disease may predispose patients to a higher risk of complications. The current patient's examination was consistent with both Wagner syndrome and regressed ROP. Versican, the protein affected in Wagner syndrome, is involved in maintaining the composition of the extracellular matrix which is important in the normal structure and function of the vitreous and vitreoretinal interface.^1^^,^^2^ As such, deficiencies of versican may predispose to excessive vitreoretinal traction, thereby increasing the risk of retinal detachment in patients with severe ROP. While the current patient's ROP severity was unknown, he manifested common findings of Wagner syndrome including high myopia, vitreous syneresis, and pigmented lattice degeneration but maintained good visual acuity in both eyes, which is often the case if the macula is not involved in regressed ROP and Wagner Syndrome.^1^^,^^2^

In addition to its role in vitreoretinal interface integrity, versican provides structural support for angiogenesis and modulates the availability of growth factors such as vascular endothelial growth factor (VEGF). Interestingly, recent studies have shown that versican, through its chondroitin sulfate chains in the versican-hyaluronan extracellular matrix, is essential for early vasculogenesis by acting as a binding scaffold for VEGF165 and Indian hedgehog.^4^ Although speculative, this raises the possibility that VCAN mutations could modulate retinal vascular development, potentially interacting with or modifying the clinical course of ROP. The current patient also manifested variants of unknown significance in CRB1 and ELOVL4, genes associated with Leber congenital amaurosis and autosomal dominant Stargardt macular degeneration, respectively. However, the patient's clinical phenotype was not consistent with either of these disorders.^10^^,^^11^

Other genetic abnormalities associated with vitreoretinal disease have been implicated in altering the presentation and natural disease course of ROP.^12^, ^13^, ^14^ For example, genetic variants associated with familial exudative vitreoretinopathy (FEVR) and telomere biology disorders have been suggested to worsen retinal ischemia in ROP, resulting in severe or atypical presentations in affected individuals.^15^, ^16^, ^17^, ^18^, ^19^, ^20^, ^21^, ^22^, ^23^, ^24^, ^25^ Indeed, evaluation for concurrent disease may be considered in patients with ROP and severe or atypical features including early-onset retinal detachment, excessive exudation, and poor treatment response.^25^ Unlike ROP alone which does not characteristically progress in adulthood, patients with ROP and concurrent vitreoretinal disease require close monitoring for disease progression throughout life.^25^ Further research is necessary to better understand the role of various coexisting vitreoretinal diseases on the course of ROP.

Previous studies have shown that rhegmatogenous retinal detachments in patients with a history of naturally regressed ROP most commonly occur between the first and third decades of life.^26^^,^^27^ The current patient has been followed until the age of 33 years with atrophic holes in both eyes but no history of retinal detachment. Because of the low prevalence of Wagner syndrome and the variability in rates of retinal detachment, it is difficult to quantify the patient's risk of retinal detachment. One study suggested that approximately 55 % of patients with Wagner syndrome have tractional retinal detachments by the age of 45 years.^1^ The risk of retinal detachment has been described more thoroughly in Stickler Syndrome, a more common hereditary vitreoretinopathy, with some recommending retinal detachment prophylaxis.^28^ There is limited data available regarding the role of retinal detachment prophylaxis in Wagner syndrome, making the management of this patient challenging.

Erosive vitreoretinopathy is believed to be an allelic variant of Wagner syndrome caused by more severe functional disruption of versican or related pathways which can be modulated by modifier genes or environmental factors.^29^ Patients with erosive vitreoretinopathy present with similar findings to Wagner syndrome in addition to an increased risk of retinal detachment, nyctalopia, and visual field loss, findings which the current patient did not manifest.^29^ Another prominent characteristic of erosive vitreoretinopathy is the presence of progressive retinal pigment epithelium atrophy which differs from the current patient who demonstrated only localized stable pigmentary changes at areas of lattice degeneration.^29^ Indeed, while the current patient's phenotype is most consistent with Wagner syndrome, erosive vitreoretinopathy should be considered in the correct clinical context.

Likewise, FEVR is an inherited condition characterized by incomplete vascularization of the peripheral retina which presents very similar to ROP.^13^^,^^30^ Unlike ROP, FEVR is associated with mutations in various genes including frizzled class receptor 4 (FZD4), Norrie disease protein (NDP), and low-density lipoprotein receptor-related protein 5 (LRP5).^30^ Furthermore, FEVR, unlike ROP, is not associated with a history of prematurity or oxygenation supplementation.^30^ While minor clinical differences exist between the 2 entities they are primarily differentiated by the aforementioned history and genetic testing.^13^

## 4 Conclusions

In this case report, we present a patient with Wagner syndrome and a history of ROP. The patient demonstrated findings characteristic of both diseases including strabismus, myopia, vitreous syneresis, lattice degeneration, and retinal holes. Given the risk of retinal detachment associated with these 2 conditions, the current patient requires life-long follow-up and monitoring for the development of further retinal breaks and associated retinal detachment. The influence of inherited retinal diseases on the natural history of ROP remains unclear. Nonetheless, it is important to consider the presence of concurrent vitreoretinal conditions in patients with a history of ROP and atypical clinical findings.

## CRediT authorship contribution statement

**Landon J. Rohowetz:** Writing – review & editing, Writing – original draft, Formal analysis, Data curation. **David W. Redick:** Writing – original draft, Formal analysis, Data curation. **Kenneth C. Fan:** Writing – original draft, Formal analysis, Data curation, Conceptualization. **Audina M. Berrocal:** Writing – review & editing, Supervision, Conceptualization.

## Patient consent

The patient consented to publication of the case in writing.

## Funding

Research to Prevent Blindness-Unrestricted Grant to BPEI (GR004596-1; New York, NY). The funding sources had no role in study design, data collection, analysis and interpretation of data, writing of the report, or in the decision to submit the article for publication.

## Declaration of competing interest

The authors declare the following financial interests/personal relationships which may be considered as potential competing interests:AMB is a consultant for Alcon, Allergan, Zeiss, Dutch Ophthalmic Research Center, Novartis, ProQR, and Oculus. KCF is a consultant for EyePoint Pharmaceuticals, RegenXBio/Abbvie, and Bayer. The following authors have no financial disclosures: LJR, DWR.
